# [Retracted] lncRNA PLAC2 activated by H3K27 acetylation promotes cell proliferation and invasion via the activation of Wnt/β-catenin pathway in oral squamous cell carcinoma

**DOI:** 10.3892/ijo.2025.5743

**Published:** 2025-03-31

**Authors:** Fubo Chen, Shengcai Qi, Xu Zhang, Jinjin Wu, Xi Yang, Raorao Wang

Int J Oncol 54: 1183-1194, 2019; DOI: 10.3892/ijo.2019.4707

Following the publication of this paper, it was drawn to the Editor's attention by a concerned reader that certain of the immunofluorescence assay data shown in Figs. 3C on p. 1189 and 4E on p. 1190 were strikingly similar to data appearing in different form in another pair of articles written by different authors at different research institutes that had already been published elsewhere prior to the submission of this paper to *International Journal of Oncology*. Given that the abovementioned data had already apparently been published previously, the Editor of *International Journal of Oncology* has decided that this paper should be retracted from the Journal. After having been in contact with the authors, they accepted the decision to retract the paper. The Editor apologizes to the readership for any inconvenience caused.

